# β-Lactamase diversity in *Pseudomonas aeruginosa*

**DOI:** 10.1128/aac.00785-24

**Published:** 2025-02-10

**Authors:** Andrew R. Mack, Andrea M. Hujer, Maria F. Mojica, Magdalena A. Taracila, Michael Feldgarden, Daniel H. Haft, William Klimke, Arjun B. Prasad, Robert A. Bonomo

**Affiliations:** 1 Department of Molecular Biology and Microbiology, Case Western Reserve University School of Medicine 12304 , Cleveland, Ohio, USA; 2 Research Service, Louis Stokes Cleveland Department of Veterans Affairs Medical Center 415208 , Cleveland, Ohio, USA; 3 Department of Medicine, Case Western Reserve University School of Medicine 12304 , Cleveland, Ohio, USA; 4 CWRU-Cleveland VAMC Center for Antimicrobial Resistance and Epidemiology (Case VA CARES) 2546 , Cleveland, Ohio, USA; 5 National Center for Biotechnology Information, National Library of Medicine, National Institutes of Health 2511 , Bethesda, Maryland, USA; 6 Department of Biochemistry, Case Western Reserve University School of Medicine 12304 , Cleveland, Ohio, USA; 7 Department of Pharmacology, Case Western Reserve University School of Medicine 12304 , Cleveland, Ohio, USA; 8 Department of Proteomics and Bioinformatics, Case Western Reserve University School of Medicine 12304 , Cleveland, Ohio, USA; 9 Clinician Scientist Investigator, Louis Stokes Cleveland Department of Veterans Affairs Medical Center 415208 , Cleveland, Ohio, USA; University of Fribourg, Fribourg, Switzerland

**Keywords:** beta-lactamases, antibiotic resistance, *Pseudomonas aeruginosa*, bioinformatics

## Abstract

*Pseudomonas aeruginosa* is a clinically important Gram-negative pathogen responsible for a wide variety of serious nosocomial and community-acquired infections. Antibiotic resistance is a major concern, as this organism has a wide variety of resistance mechanisms, including chromosomal class C (*bla*_PDC_) and D (*bla*_OXA-50_ family) β-lactamases, efflux pumps, porin channels, and the ability to readily acquire additional β-lactamases. Surveillance studies can reveal the diversity and distribution of β-lactamase alleles but are difficult and expensive to conduct. Herein, we apply a novel approach, using publicly available data derived from whole genome sequences, to explore the diversity and distribution of β-lactamase alleles across 30,452 *P*. *aeruginosa* isolates. The most common alleles were *bla*_PDC-3_, *bla*_PDC-5_, *bla*_PDC-8_, *bla*_OXA-488_, *bla*_OXA-50_, and *bla*_OXA-486_. Interestingly, only 43.6% of assigned *bla*_PDC_ alleles were encountered, and the 10 most common *bla*_PDC_ and intrinsic *bla*_OXA_ alleles represent approximately 75% of their respective total alleles, while many other assigned alleles were extremely uncommon. As anticipated, differences were observed over time and geography. Surprisingly, more distinct unassigned alleles were encountered than distinct assigned alleles. Understanding the diversity and distribution of β-lactamase alleles helps to prioritize variants for further research, select targets for drug development, and may aid in selecting therapies for a given infection.

## INTRODUCTION

*Pseudomonas aeruginosa* is a clinically important, Gram-negative pathogen responsible for a wide variety of both nosocomial and community-acquired infections, including burn, wound, respiratory tract, urinary tract, and bloodstream infections (1) and commonly harbors high levels of antibiotic resistance (2). Carbapenem-resistant *P. aeruginosa* (CRPa) is considered a “high-priority pathogen” by the World Health Organization (WHO) (3), and the Centers for Disease Control and Prevention (CDC) has declared multidrug-resistant (MDR) *P. aeruginosa* a “serious threat,” responsible for 2,700 deaths and $767 million in healthcare costs in the United States in 2017 (4). Likely driven by the COVID-19 pandemic, rates of hospital-onset MDR *P. aeruginosa* infections increased 32% from 2019 to 2020 (5).

Unfortunately, treatment of these diverse infections is complicated by a wide array of intrinsic, acquired, and mutational mechanisms—including multiple β-lactamases, alterations in outer membrane porins and efflux pumps, and target modification—that provide resistance to large numbers of antibiotics (2).

While many recent analyses have examined the prevalence of β-lactamases in *P. aeruginosa* (6–12), such studies often utilize a relatively limited sample size (both in quantity and distribution), have a specific and somewhat limited focus (e.g., carbapenemase genes), look only at data collected two or more years prior to publication, and rarely utilize whole-genome sequencing (WGS) as a means of exploring the full diversity of the resistome. A database of amino acid variants associated with assigned *bla*_PDC_ alleles has been developed (13), along with a *P. aeruginosa* diversity panel, which captures a wide variety of β-lactamase alleles and other resistance determinants (14), but neither provides dynamic snapshots of allelic diversity.

Given the relative lack of broad surveys of β-lactamase alleles, we set out to gain a better understanding of the genetic landscape of *P. aeruginosa*. Herein, we analyzed data on more than 30,000 isolates found in the National Center for Biotechnology Information (NCBI) Pathogen Detection databases to gain insights into the *P. aeruginosa* β-lactamase resistome. Querying and analyzing a large, rich data set allow for the examination of a wide array of variables and provide additional dimensions over which to analyze the data. Furthermore, the NCBI Pathogen Detection pipeline provides data in near real time (15), potentially allowing changes in patterns of resistance genes to be examined quickly and efficiently. Understanding the genetic diversity of β-lactamases in *P. aeruginosa* will help drive innovation in multiple ways: at the basic science level, it will help prioritize β-lactamase variants to study biochemically and microbiologically to understand changing resistance mechanisms; at the translational level, it will help select targets for the design, optimization, and testing of new β-lactam antibiotics and β-lactamase inhibitors; and at the clinical level, it can aid in the selection of the most promising therapies for a given infection.

## RESULTS

### Overview of the data set

As of 9 September 2024, the NCBI Microbial Browser for Identification of Genetic and Genomic Elements (MicroBIGG-E) database covered 31,061 *P*. *aeruginosa* isolates. After filtering for isolates encoding at least one full-length, high-quality *bla*_PDC_ allele and one full-length, high-quality intrinsic *bla*_OXA-50_ family allele, and deduplicating at the isolate level, the final data set contained 30,452 isolates encoding a total of 73,147 distinct β-lactamase (*bla*) genes (Table S1).

Unsurprisingly, *bla*_PDC_ (30,842 genes) and intrinsic *bla*_OXA_ (*bla*_OXA-50_ family members; 30,571 genes) were the most commonly encountered *bla* gene families, collectively accounting for 84.0% of all *bla* alleles in the data set. Members of 9 acquired *bla*_OXA_ families and 28 other acquired *bla* gene families were encountered in five or more isolates each, representing a total of 10,792 acquired genes. For the purposes of this analysis, “assigned” refers to a distinct *bla* allele that has been designated with an identifying number (e.g., *bla*_PDC-3_); “unassigned” *bla* alleles do not have a specific number attributed to that *bla* gene. Collectively, 531 distinct, assigned *bla* alleles were present, including 254 distinct, assigned *bla*_PDC_ alleles and 63 distinct, assigned, intrinsic *bla*_OXA_ alleles. A total of 3,160 unassigned *bla* alleles were present, including 1,473 unassigned *bla*_PDC_ genes (622 distinct alleles) and 1,369 unassigned, intrinsic *bla*_OXA_ genes (262 distinct alleles) (Table S2).

### Origin of isolates

#### Geographic

**Fig 1:**
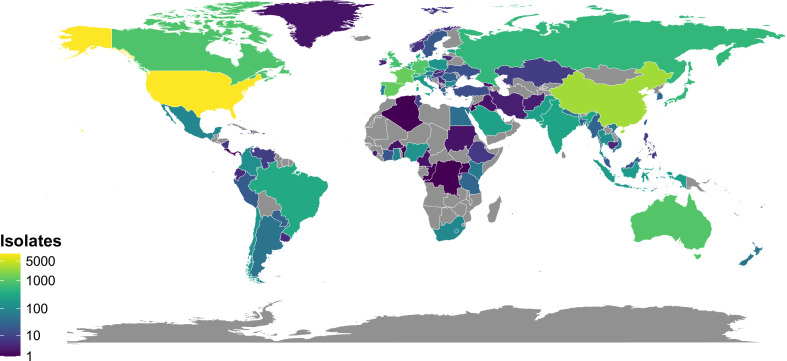
Geographic origin of the isolates used in the analysis. Geographic origin was unavailable for 4,774 of 30,452 isolates. Gray indicates that no isolates were analyzed from a given country/region. The map was generated using the ggplot2 package for R with the default world map data.

The data set contains one or more isolates from 99 countries and at least 400 from each of the eight regions examined (Fig. 1; Fig. S1; Table S3). The United States and China were the largest contributors (30.2% and 9.4% of isolates, respectively), with 39 countries contributing 50 or more isolates each (Table S3). North America, Europe, Central Asia, and East & Southeast Asia contributed the most isolates (Table S3; Fig. S1). Unsurprisingly, higher- and middle-income countries tend to be better represented.

#### Collection date

Both historic and contemporary isolates are included, but the vast majority have been collected in recent years as large-scale collection, and sequencing has become more frequent and affordable. A total of 495 isolates were collected before the year 2000, and an average of 1,916 isolates were collected annually from 2015 to 2023 (Table S4).

### Overview of β-lactamase genes

The three most commonly assigned *bla*_PDC_ alleles are *bla*_PDC-3_ (5,670 isolates, 18.6%), *bla*_PDC-5_ (4,269 isolates, 14.0%), and *bla*_PDC-8_ (3,577 isolates, 11.7%). An additional 12 alleles occur in 1.0%–10.0% of isolates and 9 in 0.5%–1.0% of isolates (Table 1). Contrastingly, 171 alleles occur in 10 or fewer isolates, including 70 found in only a single isolate (Table S5). Unassigned alleles follow similar patterns, with just one distinct allele (of 622 total) occurring in 0.10% or more of isolates (NCBI Protein Accession Number HDQ8152859.1 [281 isolates, 0.92%]) and 614 distinct alleles occurring in five or fewer isolates, including 513 occurring only once (Table S6).

**TABLE 1:** Intrinsic *bla*_PDC_ (left) and intrinsic *bla*_OXA_ (right) alleles occurring in 100 or more isolates^*a*^

Allele	Isolates	Percentage	Rank	Allele	Isolates	Percentage	Rank
*bla*_PDC-3_	5,670	18.62	1	*bla*_OXA-488_	4,672	15.34	1
*bla*_PDC-5_	4,269	14.02	2	*bla*_OXA-50_	3,821	12.55	2
*bla*_PDC-8_	3,577	11.75	3	*bla*_OXA-486_	3,608	11.85	3
*bla*_PDC-1_	2,154	7.07	4	*bla*_OXA-494_	3,126	10.27	4
*bla*_PDC-35_	2,137	7.02	5	*bla*_OXA-395_	2,543	8.35	5
*bla*_PDC-19a_	1,539	5.05	6	*bla*_OXA-396_	2,161	7.10	6
*bla*_PDC-16_	1,145	3.76	7	*bla*_OXA-847_	1,524	5.00	7
*bla*_PDC-34_	906	2.98	8	*bla*_OXA-904_	845	2.77	8
*bla*_PDC-24_	850	2.79	9	*bla*_OXA-905_	768	2.52	9
*bla*_PDC-11_	656	2.15	10	*bla*_OXA-846_	724	2.38	10
*bla*_PDC-15_	464	1.52	11	*bla*_OXA-848_	669	2.20	11
*bla*_PDC-31_	396	1.30	12	*bla*_OXA-851_	588	1.93	12
*bla*_PDC-12_	361	1.19	13	*bla*_OXA-1028_	552	1.81	13
*bla*_PDC-30_	331	1.09	14	*bla*_OXA-1032_	485	1.59	14
*bla*_PDC-36_	330	1.08	15	*bla*_OXA-1035_	452	1.48	15
*bla*_PDC-37_	248	0.81	16	*bla*_OXA-914_	420	1.38	16
*bla*_PDC-14_	240	0.79	17	*bla*_OXA-1127_	234	0.77	17
*bla*_PDC-103_	215	0.71	18	*bla*_OXA-1034_	212	0.70	18
*bla*_PDC-6_	169	0.55	19	*bla*_OXA-1026_	154	0.51	19
*bla*_PDC-121_	162	0.53	20	*bla*_OXA-901_	153	0.50	20
*bla*_PDC-39_	161	0.53	21	*bla*_OXA-1014_	131	0.43	21
*bla*_PDC-60_	161	0.53	21	*bla*_OXA-1030_	126	0.41	22
*bla*_PDC-22_	158	0.52	22	*bla*_OXA-1033_	119	0.39	23
*bla*_PDC-46_	153	0.50	23	*bla*_OXA-906_	118	0.39	24
*bla*_PDC-59_	123	0.40	24				
*bla*_PDC-120_	120	0.39	25				
*bla*_PDC-66_	118	0.39	26				
*bla*_PDC-71_	115	0.38	27				
*bla*_PDC-98_	110	0.36	28				

^*a*^ Percentage represents the percentage of isolates encoding a specific allele.

Among intrinsic *bla*_OXA_ alleles, the four most common are *bla*_OXA-488_ (4,672 isolates, 15.3%), *bla*_OXA-50_ (3,821 isolates, 12.5%), *bla*_OXA-486_ (3,608 isolates, 11.8%), and *bla*_OXA-494_ (3,126 isolates, 10.3%). An additional 12 alleles occur in 1.0%–10.0% and 4 in 0.5%–1.0% of isolates (Table 1). In contrast to *bla*_PDC_, only 45 alleles occur in 10 or fewer isolates and just 6 in only a single isolate (Table S5). Three distinct unassigned alleles occur in 0.10% or more of isolates (RefSeq [16] protein accession number WP_058176185.1 [49 isolates, 0.16%], WP_034047652.1 [31 isolates, 0.10%], and WP_034040414.1 [30 isolates, 0.10%]), while 349 appear in five or fewer isolates, including 165 in only a single isolate (Table S6).

Acquired *bla* alleles represent a total of 10,494 genes, consisting of 174 distinct, assigned alleles across 10 *bla*_OXA_ families and 27 other *bla* gene families found in five or more isolates. The most common acquired gene was *bla*_VIM_ (2,454 isolates), and an additional 12 were found in 100 or more isolates each: *bla*_OXA-10_ family, *bla*_OXA-2_ family, *bla*_GES_, *bla*_IMP_, *bla*_KPC_, *bla*_NDM_, *bla*_CARB_, *bla*_OXA-1_ family, *bla*_TEM_, *bla*_VEB_, *bla*_PER_, and *bla*_PAC_ (Table S7). The seven most commonly acquired alleles, each occurring in 1.0% or more of isolates, were *bla*_VIM-2_ (1,703 alleles, 5.6%), *bla*_OXA-2_ (1,240 isolates, 4.1%), *bla*_OXA-10_ (955 isolates, 3.1%), *bla*_KPC-2_ (822 isolates, 2.7%), *bla*_NDM-1_ (714 isolates, 2.3%), *bla*_CARB-2_ (479 isolates, 1.6%), and *bla*_GES-5_ (324 isolates, 1.1%) (Table 2). Collectively, members of three *bla*_OXA_ families appeared in 1.0% or more of isolates: the *bla*_OXA-10_ family (1,493 isolates, 4.9%), the *bla*_OXA-2_ family (1,319 isolates, 4.3%), and the *bla*_OXA-1_ family (477 isolates, 1.6%) (Table S7).

**TABLE 2:** Acquired *bla* alleles found in 100 or more isolates^*a*^

Allele	*bla*_OXA_ family	Isolates	Percentage
*bla*_VIM-2_		1,703	5.59
*bla*_OXA-2_	*bla*_OXA-2_	1,240	4.07
*bla*_OXA-10_	*bla*_OXA-10_	955	3.14
*bla*_KPC-2_		822	2.70
*bla*_NDM-1_		714	2.34
*bla*_CARB-2_		479	1.57
*bla*_GES-5_		324	1.06
*bla*_IMP-7_		227	0.75
*bla*_VEB-9_		224	0.74
*bla*_OXA-4_	*bla*_OXA-1_	221	0.73
*bla*_GES-9_		221	0.73
*bla*_VIM-1_		214	0.70
*bla*_PER-1_		213	0.70
*bla*_VIM-80_		209	0.69
*bla*_TEM-1_		205	0.67
*bla*_TEM-116_		187	0.61
*bla*_IMP-1_		181	0.59
*bla*_VIM-4_		163	0.54
*bla*_GES-1_		162	0.53
*bla*_OXA-246_	*bla*_OXA-10_	129	0.42
*bla*_OXA-1_	*bla*_OXA-1_	123	0.40
*bla*_GES-20_		121	0.40
*bla*_GES-19_		117	0.38
*bla*_PAC-1_		114	0.37
*bla*_OXA-101_	*bla*_OXA-10_	104	0.34
*bla*_IMP-13_		103	0.34

^*a*^ Percentage represents the percentage of isolates encoding a specific allele.

### Allele frequency by region

**Fig 2:**
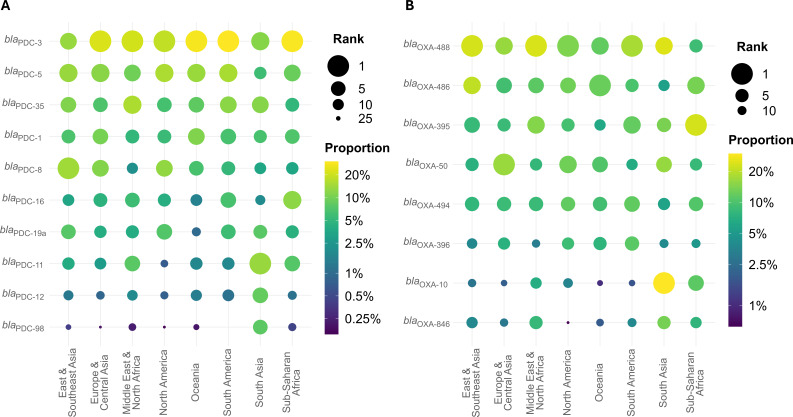
Intrinsic alleles by region. Rank and proportion of the five most common (**A**) *bla*_PDC_ and (**B**) *bla*_OXA_ alleles in isolates from each region across all regions. Color indicates the proportion of isolates containing a given allele, and size indicates the frequency rank of an allele in each region.

Among *bla*_PDC_, the most common allele is *bla*_PDC-3_ in all regions except East & Southeast Asia (third most common after *bla*_PDC-8_ and *bla*_PDC-5_) and South Asia (second most common after *bla*_PDC-11_). Uniformity across regions is high, with *bla*_PDC-1_, *bla*_PDC-3_, *bla*_PDC-5_, *bla*_PDC-8_, *bla*_PDC-19a_, and *bla*_PDC-35_ generally among the most common alleles in all regions. The most region-specific alleles are *bla*_PDC-16_ (second most common in Sub-Saharan Africa but 5th to 13th most common elsewhere), *bla*_PDC-98_ (fifth most common in South Asia and no higher than 16th most common in any other region), and *bla*_PDC-97_ (seventh most common in Oceania but only 19th to 36th most common in the three other regions it appears) (Fig. 2A; Table S8).

Among intrinsic *bla*_OXA_, *bla*_OXA-488_ is the most or second most common in all regions except Sub-Saharan Africa (fourth most common), while *bla*_OXA-486_ and *bla*_OXA-395_ are among the three most common in six and five of the eight regions, respectively. The most region-specific alleles are *bla*_OXA-914_ (9th most common in North America, but 14th to 21st in the six other regions it is found), *bla*_OXA-905_ (7th most common in Europe & Central Asia, but 12th to 28th in the other regions it is found), and *bla*_OXA-1029_ (9th most common in South Asia, but 14th to 33rd in the other regions it is found) (Fig. 2B; Table S9).

Interestingly, the prevalence of acquired alleles varies widely by region. Acquired *bla*_OXA-10_ family genes range in prevalence from 1.9% of isolates in Oceania to 30.7% in South Asia, *bla*_OXA-2_ family genes from 0.9% in South Asia to 8.0% in North America, *bla*_VIM_ genes from 1.5% in Oceania to 21.0% in Sub-Saharan Africa, *bla*_IMP_ genes from 1.8% in North America to 8.2% in Oceania, *bla*_GES_ genes from 1.9% in Europe & Central Asia to 12.3% in South Asia, *bla*_KPC_ genes from 0.1% in Oceania and Europe & Central Asia to 17.5% in South America, and *bla*_NDM_ genes from 0.9% in Europe & Central Asia to 14.9% in Sub-Saharan Africa (Table S10).

### Allele frequency with time

**Fig 3:**
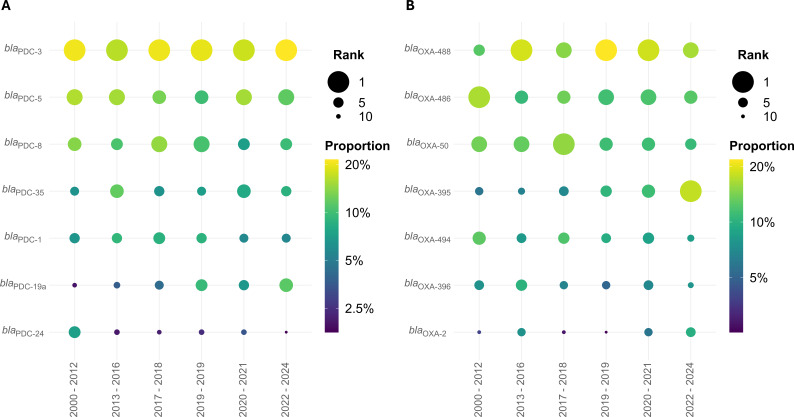
Intrinsic alleles by collection period. Rank and proportion of the five most common (**A**) *bla*_PDC_ and (**B**) *bla*_OXA_ alleles in isolates from each collection period across all periods. Color indicates the proportion of isolates containing a given allele, and size indicates the frequency rank of an allele in each period.

The most common *bla*_PDC_ allele overall (*bla*_PDC-3_) remains the most common, and the second and third overall (*bla*_PDC-5_ and *bla*_PDC-8_) remain among the top four most common across all periods since 2000, demonstrating the stability of the most common alleles over time. Contrastingly, *bla*_PDC-19a_ increased from 10th most common in 2000–2012 to 3rd most common in 2022–2024, and *bla*_PDC-24_ decreased from 4th most common in 2000–2012 to 9th to 12th most common in all subsequent periods (Fig. 3A; Table S11).

Among *bla*_OXA_ alleles, the six most common alleles overall remain relatively stable over time, ranking among the six most common alleles in all time periods since 2000. Within this, *bla*_OXA-395_ increased from sixth most common through 2016 to third most common in 2020–2021 and most common in 2022–2024 (Fig. 3B; Table S12).

### Allele combinations

Combinations of β-lactamase alleles are relatively well-distributed, with 21 allele combinations occurring in 1.0% or more of isolates, the five most common of which are *bla*_PDC-5_/*bla*_OXA-494_ (1,014 isolates, 3.3%), *bla*_PDC-34_/*bla*_OXA-488_ (822 isolates, 2.7%), *bla*_PDC-5_/*bla*_OXA-396_ (820 isolates, 2.7%), *bla*_PDC-3_/*bla*_OXA-486_ (820 isolates, 2.7%), and *bla*_PDC-8_/*bla*_OXA-50_ (795 isolates, 2.6%) (Table 3). Contrastingly, 1,975 combinations occur in 10 or fewer isolates, including 1,165 in only a single isolate (Table S13). Only three combinations occurring in 1.0% or more of isolates contain an acquired β-lactamase: *bla*_PDC-3_/*bla*_OXA-395_/*bla*_VIM-2_ (13th most common; 528 isolates, 1.7%), *bla*_PDC-8_/*bla*_OXA-486_/*bla*_KPC-2_ (18th most common; 367 isolates, 1.2%), and *bla*_PDC-19a_/*bla*_OXA-488_/*bla*_NDM-1_ (21st most common; 306 isolates, 1.0%) (Table S13), underscoring the relative infrequency of acquired *bla* alleles in *P. aeruginosa*. Considering only intrinsic alleles, the most common combinations are *bla*_PDC-35_/*bla*_OXA-488_ (2,072 isolates, 6.8%), *bla*_PDC-3_/*bla*_OXA-395_ (1,221 isolates, 4.0%), *bla*_PDC-3_/*bla*_OXA-486_ (1,185 isolates, 3.9%), *bla*_PDC-5_/*bla*_OXA-494_/ (1,138 isolates, 3.7%), and *bla*_PDC-5_/*bla*_OXA-396_ (928 isolates, 3.0%) (Table S14).

**TABLE 3:** Allele combinations occurring in 100 or more isolates

Combination of alleles	Isolates	Percentage
*bla*_OXA-494,_ *bla*_PDC-5_	1,014	3.33
*bla*_OXA-488,_ *bla*_PDC-34_	822	2.70
*bla*_OXA-396,_ *bla*_PDC-5_	820	2.69
*bla*_OXA-486,_ *bla*_PDC-3_	820	2.69
*bla*_OXA-50,_ *bla*_PDC-8_	795	2.61
*bla*_OXA-50,_ *bla*_PDC-3_	720	2.36
*bla*_OXA-486,_ *bla*_PDC-24_	688	2.26
*bla*_OXA-847,_ *bla*_PDC-1_	634	2.08
*bla*_OXA-50,_ *bla*_PDC-1_	625	2.05
*bla*_OXA-396,_ *bla*_PDC-8_	617	2.03
*bla*_OXA-905,_ *bla*_PDC-8_	606	1.99
*bla*_OXA-494,_ *bla*_PDC-3_	546	1.79
*bla*_OXA-395,_ *bla*_PDC-3,_ *bla*_VIM-2_	528	1.73
*bla*_OXA-488,_ *bla*_PDC-35_	519	1.70
*bla*_OXA-494,_ *bla*_PDC-15_	434	1.43
*bla*_OXA-848,_ *bla*_PDC-16_	434	1.43
*bla*_OXA-50,_ *bla*_PDC-5_	415	1.36
*bla*_KPC-2,_ *bla*_OXA-486,_ *bla*_PDC-8_	367	1.21
*bla*_OXA-904,_ *bla*_PDC-3_	359	1.18
*bla*_OXA-847,_ *bla*_PDC_	332	1.09
*bla*_NDM-1,_ *bla*_OXA-488,_ *bla*_PDC-19a_	306	1.00
*bla*_GES-5,_ *bla*_OXA-488,_ *bla*_PDC-35_	281	0.92
*bla*_OXA-488,_ *bla*_PDC-19a_	278	0.91
*bla*_OXA-395,_ *bla*_PDC-3_	259	0.85
*bla*_OXA-395,_ *bla*_PDC-36_	258	0.85
*bla*_OXA-2,_ *bla*_OXA-914,_ *bla*_PDC-5_	247	0.81
*bla*_OXA-50,_ *bla*_PDC-14_	233	0.77
*bla*_OXA-1035,_ *bla*_PDC-19a_	232	0.76
*bla*_OXA-486,_ *bla*_PDC-5_	225	0.74
*bla*_OXA,_ *bla*_PDC-5_	202	0.66
*bla*_OXA-488,_ *bla*_PDC-35,_ *bla*_VIM-2_	198	0.65
*bla*_OXA,_ *bla*_PDC-3_	198	0.65
*bla*_GES-9,_ *bla*_OXA-10,_ *bla*_OXA-395,_ *bla*_PDC-19a,_ *bla*_VIM-80_	186	0.61
*bla*_OXA-486,_ *bla*_PDC-8_	185	0.61
*bla*_OXA-486,_ *bla*_PDC-1_	184	0.60
*bla*_OXA-494,_ *bla*_PDC-8_	174	0.57
*bla*_OXA-851,_ *bla*_PDC-5_	174	0.57
*bla*_IMP-7,_ *bla*_OXA-2,_ *bla*_OXA-846,_ *bla*_PDC-11_	167	0.55
*bla*_OXA-488,_ *bla*_PDC-30_	164	0.54
*bla*_OXA-1034,_ *bla*_PDC-3_	155	0.51
*bla*_OXA-846,_ *bla*_PDC-11_	149	0.49
*bla*_OXA-395,_ *bla*_PDC-30_	147	0.48
*bla*_OXA-494,_ *bla*_PDC_	147	0.48
*bla*_NDM-1,_ *bla*_OXA-395,_ *bla*_PDC-16_	146	0.48
*bla*_OXA-1028,_ *bla*_PDC-8_	142	0.47
*bla*_OXA-488,_ *bla*_PDC-37_	141	0.46
*bla*_OXA-4,_ *bla*_OXA-486,_ *bla*_PDC-3,_ *bla*_VIM-2_	139	0.46
*bla*_OXA-904,_ *bla*_PDC-5_	138	0.45
*bla*_OXA-1032,_ *bla*_PDC-16_	130	0.43
*bla*_OXA-2,_ *bla*_OXA-488,_ *bla*_PDC-35_	128	0.42
*bla*_OXA-1032,_ *bla*_PDC-22_	127	0.42
*bla*_OXA-1127,_ *bla*_PDC-5_	120	0.39
*bla*_OXA-50,_ *bla*_PDC-31_	120	0.39
*bla*_OXA-1028,_ *bla*_PDC-5_	119	0.39
*bla*_OXA-851,_ *bla*_PDC-8_	115	0.38
*bla*_OXA-486,_ *bla*_PDC_	113	0.37
*bla*_OXA-1028,_ *bla*_PDC-3_	111	0.36
*bla*_OXA-906,_ *bla*_PDC-59_	111	0.36
*bla*_OXA-494,_ *bla*_PDC-71_	108	0.35
*bla*_OXA-1033,_ *bla*_PDC-3_	105	0.34
*bla*_OXA-914,_ *bla*_PDC-5_	104	0.34

### Carbapenemases

Unlike *Acinetobacter baumannii*, the intrinsic *bla*_OXA_ alleles of *P. aeruginosa* are not typically associated with carbapenemase activity. Acquired carbapenemase genes, however, are present in 5,449 isolates (17.9%). The most common families of acquired carbapenemases are *bla*_VIM_ (2,454 isolates, 8.1%), *bla*_IMP_ (917 isolates, 3.0%), *bla*_KPC_ (887 isolates, 2.9%), *bla*_NDM_ (718 isolates, 2.4%), and *bla*_GES_ (467 isolates, 1.5%), with seven additional gene families and two acquired *bla*_OXA_ families found across 165 isolates (Table S15). A total of 11 distinct, assigned carbapenemase alleles occurred in 100 or more isolates each: 4 of 27 total encountered *bla*_VIM_ carbapenemase alleles, 3 of 42 total encountered *bla*_IMP_ carbapenemase alleles, 2 of 7 total encountered *bla*_GES_ carbapenemase alleles, 1 of 6 total encountered *bla*_KPC_ carbapenemase alleles, and 1 of 4 total encountered *bla*_NDM_ carbapenemase alleles (Table S15).

Concerningly, metallo-β-lactamase genes with carbapenemase activity are present in 4,142 isolates (13.6%), many of which are likely to provide resistance to large swaths of the β-lactam armamentarium. The most common metallo-β-lactamases encountered were *bla*_VIM-2_ (1,703 isolates, 5.6%), *bla*_NDM-1_ (714 isolates, 2.3%), *bla*_IMP-7_ (227 isolates, 0.7%), *bla*_VIM-1_ (214 isolates, 0.7%), *bla*_VIM-80_ (209 isolates, 0.7%), *bla*_IMP-1_ (181 isolates, 0.6%), *bla*_VIM-4_ (163 isolates, 0.5%), and *bla*_IMP-12_ (103 isolates, 0.3%) (Table S15).

**Fig 4:**
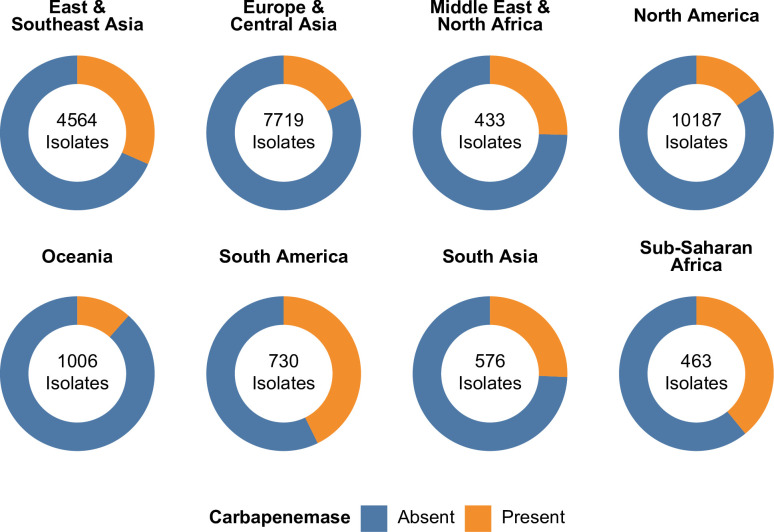
Proportion of isolates encoding carbapenemase genes by region. A total of 5,606 isolates (19.6%) encode carbapenemase genes.

Alarmingly, carbapenemase genes appear to have become more common over time, increasing from 8.4% of isolates collected in 2000–2012 to 39.8% of isolates collected in 2022–2024 (Table S16). The overall proportion of isolates containing carbapenemase genes ranges from 11.5% in Oceania to 42.7% in South America (Table S17; Fig. 4). Interestingly, isolates without collection date or collection location metadata are far less likely to encode carbapenemase alleles (7.7% and 4.1%, respectively), but the reasons behind this are not clear.

### Breadth of allelic diversity

As isolates were not collected under a uniform protocol, both over- and under-representation of alleles are likely. To address this, we “deduplicated” and examined the binary presence of distinct alleles within each group, which helps to mitigate the impact of successful clones and outbreaks and amplify the signal of widespread but low-frequency alleles.

#### MLST and pathogen detection SNP cluster frequency

Multilocus sequence typing (MLST) (17) and whole genome multilocus sequence typing (wgMLST) (18) are common approaches to determining the relatedness of bacterial isolates. The former utilizes a curated set of typically seven slowly evolving housekeeping genes to determine allele variants and assign a sequence type (ST), while the latter utilizes a much larger set of genes extracted from WGS data to determine the relatedness of isolates (19). NCBI Pathogen Detection generates wgMLST-based nearest-neighbor clusters of very closely related isolates, using a 25-allele cutoff (https://www.ncbi.nlm.nih.gov/pathogens/pathogens_help/#data-processing-clustering), which are given accessions starting with “PDS” (standing for Pathogen Detection Single nucleotide polymorphism [SNP]) and herein referred to as PDS clusters. Note that PDS clusters can change over time and are not archived, so the exact accessions mentioned below pertain only to the specific snapshot of data used in this analysis. The general patterns and trends should hold true across releases.

We found 1,863 distinct STs by MLST. The most common were ST235 (2,118 isolates, 7.0%) and ST111 (1,142 isolates, 3.8%), and nine others contain 2.0% or more of isolates: ST253, ST244, ST308, ST395, ST17, ST274, ST179, ST357, and ST155 (Table S18). By PDS, the data set contains 3,288 distinct clusters, of which PDS000112090.129 is the most common (640 isolates, 2.1%) with two other clusters occurring in 1.0% or more of isolates: PDS000095640.35 and PDS000012474.54. (Table S19). An additional 12,153 isolates were not assigned to a cluster, suggesting they are not closely related to any other isolate.

Changes in the frequency of STs were observed over time and geography. Temporally, ST235 is among the 2 most common across all periods, ST111 is 2nd through 6th most common across all periods, and ST253 is between 5th and 11th most common across all periods, none of which appear to be trending. Several STs exhibit dramatic variability between periods, perhaps associated with outbreaks or changes in the population structure over time: ST146 was the 9th most common in 2000–2012, 18th in 2013–2016, no higher than 28th in any other period, and was not observed in 2022–2024; ST316 was the 2nd most common in 2020–2021 but no higher than 27th most common in any other period, and ST1076 was the 8th most common in 2000–2012 but no higher than 21st most common in any other period, and ST1203 was the 3rd most common in 2022–2024 but no higher than 36th in any other period and not observed in 2000–2012 (Table S20). Regionally, ST235 is among the 2 most common in all regions except Sub-Saharan Africa (5th most common), ST111 is 2nd to 5th most common across six regions, but 16th in South Asia and 21st in East and Southeast Asia, and ST253 is between 4th and 15th most common across all regions. Among the most regionally variable STs, ST463 is the 2nd most common in East and Southeast Asia but no higher than 16th in any other region and not found in four regions; ST357 is the 26th most common in North America but 1st to 9th most common in all other regions; ST395 is the third most common in Europe and Central Asia but no higher than 12th most common in any other region and not found in two regions; and ST649 is the sixth most common in Oceania but no more than 16th most common in any other region and not found in four regions (Table S21).

An overview of allele frequency when the data are examined for the presence of individual alleles by ST and PDS clusters to better account for closely related isolates is provided in the supplemental text and Tables S22 and 23.

#### MLST and PDS association with intrinsic alleles

Given that sequence types and PDS clusters both serve to group related alleles, the question of whether certain STs and clusters are associated with specific alleles or whether certain alleles are associated with specific clusters (as might be expected if alleles are primarily disseminated in a clonal manner) naturally arises.

Looking at MLST, 1,574 (89.4%) of 1,761 STs associated with an assigned *bla*_PDC_ allele correspond to a single allele, 138 (7.8%) to two alleles, 26 (1.5%) to three alleles, and 23 (1.3%) to four or more alleles, including ST235 (2,110 isolates corresponding to nine distinct alleles) and ST308 (754 isolates corresponding to 10 distinct alleles). Interestingly, intrinsic *bla*_OXA_ alleles are more strongly associated with STs—1,605 (96.3%) of 1,667 STs associated with assigned intrinsic *bla*_OXA_ alleles correspond to a single allele, 57 (including ST235) to two alleles, and 5 to three alleles (Table S24). Compared to STs, PDS clusters associated with assigned *bla*_PDC_ alleles generally correspond to fewer alleles, with the vast majority of clusters (including the 10 most common, encompassing a combined 2,840 isolates) encoding just a single *bla*_PDC_ allele, 46 clusters (1.4%) encoding two alleles, 1 cluster encoding three alleles, and 1 cluster (PDS000064916.3, consisting of 24 isolates) encoding four alleles. Clusters associated with assigned *bla*_OXA_ alleles again correspond to far fewer alleles, with 3,141 (99.7%) of 3,150 clusters corresponding to a single allele and 9 clusters (0.3%) corresponding to two alleles (Table S25).

While STs and clusters are often associated with low numbers of distinct alleles, the opposite is not true. Among the 245 assigned *bla*_PDC_ alleles associated with defined STs, the most widespread are *bla*_PDC-3_ (542 STs, 29.1%), *bla*_PDC-5_ (367 STs, 19.7%), *bla*_PDC-9_ (113 STs, 6.1%), and *bla*_PDC-1_ (105 STs, 5.6%), with a total of 52 alleles found in five or more STs each and only 120 alleles (49.0%) with a single ST. Of the 61 assigned *bla*_OXA_ alleles associated with defined STs, the most widespread are *bla*_OXA-494_ (304 STs, 16.3%), *bla*_OXA-50_ (268 STs, 14.4%), and *bla*_OXA-486_ (232 STs, 12.5%), with a total of 40 alleles (65.6%) corresponding to five or more STs and only 10 alleles (16.4%) to single ST each (Table S26). Similar patterns emerge with clusters: of the 156 assigned *bla*_PDC_ alleles associated with clusters, the most widespread are *bla*_PDC-3_ (622 clusters, 18.9%), *bla*_PDC-5_ (492 clusters, 15.0%), *bla*_PDC-8_ (350 clusters, 10.6%), *bla*_PDC-1_ (248 clusters, 7.5%), *bla*_PDC-35_ (244 clusters, 7.4%), *bla*_PDC-19a_ (127 clusters, 3.9%), *bla*_PDC-16_ (125 clusters, 3.8%), and *bla*_PDC-24_ (102 clusters, 3.1%), with a total of 46 alleles (29.5%) corresponding to five or more clusters and 70 alleles (44.9%) to a single cluster. Among the 52 assigned, intrinsic *bla*_OXA_ alleles associated with clusters, the most widespread are *bla*_OXA-488_ (528 clusters, 16.1%), *bla*_OXA-50_ (429 clusters, 13.0%), *bla*_OXA-494_ (400 clusters, 12.2%), *bla*_OXA-486_ (372 clusters, 11.3%), *bla*_OXA-396_ (234 clusters, 7.1%), *bla*_OXA-395_ (192 clusters, 5.8%), and *bla*_OXA-847_ (159 clusters, 4.8%), with a total of 31 alleles (59.6%) corresponding to five or more clusters and just 9 alleles (17.3%) to a single cluster (Table S27).

#### MLST and PDS granularity

Unsurprisingly, the higher resolution of wgMLST means PDS clusters provide more granularity than STs, resulting in a more diverse (and likely more representative) deduplicated data set. This difference in granularity is exemplified by the tendency of a single ST to correspond to many clusters, while the opposite is generally not true. Notably, ST235 (encompassing 7.0% of isolates) corresponds to 244 PDS clusters, and ST244 (encompassing 2.9% of isolates) corresponds to 122 PDS clusters, while 59 additional STs correspond to 10 or more clusters each, and 1,256 STs (67.4%) correspond to only a single cluster. Conversely, just 1 of 3,288 PDS clusters corresponds to three STs, 13 (0.5%) correspond to two STs, and 3,151 (95.8%) correspond to only a single ST (Table S28).

#### BioProject

BioProjects link isolates collected as part of the same research projects or studies, suggesting that it may be possible for isolates within them to be either closely related or selected based on similar phenotypic criteria, potentially impacting the results of the analysis. A total of 1,275 BioProjects were included in the data set, the largest of which vary greatly by region, ranging from 2,083 isolates (North America) to 69 isolates (Middle East and North Africa). Interestingly, the median number of isolates belonging to a BioProject is one or two across all regions, suggesting the results are not overrun with large BioProjects in general (Table S29).

Examining the presence of alleles by BioProject has minimal impact on overall allele frequency. The two most common *bla*_PDC_ alleles, *bla*_PDC-3_ (455 BioProjects) and *bla*_PDC-5_ (377 BioProjects), remain unchanged, and none of the 10 most common alleles shift by more than one rank. Among intrinsic *bla*_OXA_, the two most common alleles, *bla*_OXA-488_ (470 BioProjects) and *bla*_OXA-50_ (455 BioProjects), remain unchanged, and none of the 10 most common alleles shift by more than three ranks (Table S30).

### Comparing *Pseudomonas* alleles

Combining the sequences of assigned alleles present in the Reference Gene Catalog with those of unassigned alleles present in the data set provides a substantial snapshot into the diversity of β-lactamase alleles in *P. aeruginosa*.

### Amino acid conservation

#### Conservation among *bla*_PDC_ variants

**Fig 5:**
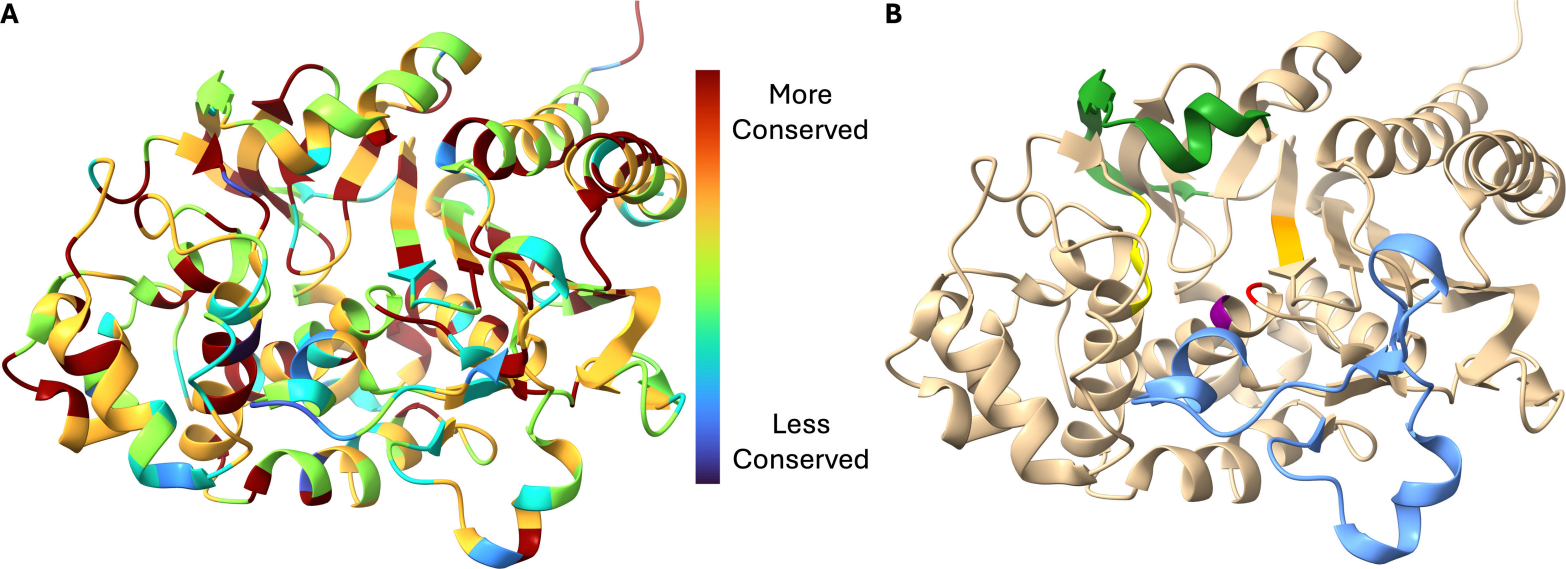
Residue-by-residue conservation of *bla*_PDC_ alleles. (**A**) Color-coded conservation of amino acid residues. (**B**) Important regions and motifs of class C β-lactamases, including the catalytic serine (S64; red), general base lysine (K67; purple), general base tyrosine (Y150, as part of the YSN motif; yellow), the Ω-loop (blue), the R2-loop (forest green), and the KTG motif (golden orange). Visualized on the crystal structure of PDC-1 using PDB ID: 4GZB (23).

Determining amino acid conservation among the distinct *bla*_PDC_ alleles present in the data set and using the results to color code an experimentally determined crystal structure provide a residue-by-residue overview of conservation in PDC (Fig. 5A). For reference, Fig. 5B highlights important regions and residues of these class C β-lactamases, including the catalytic serine (S64), the general base lysine (K67), the YSN motif (containing the general base Y150), the Ω-loop (residues 188–221), the R2-loop (residues 289–307), and the KTG motif (containing K315) (20–22).

As anticipated, the essential class C β-lactamase catalytic residues and characteristic motifs (S64-V65-S66-K67, Y150-S151-N152, and K315-T316-G317 by the [SANC] amino acid numbering convention) (20, 21) are highly conserved, as are several important residues in the C_3_/C_4_ carboxylate recognition region. The core portions of α-helices are also generally well conserved. Regions of lower conservation are scattered throughout, including portions of both the Ω-loop and R2-loop, regions known to play major roles in determining substrate specificity and the ability of variant enzymes to hydrolyze newer, larger, or otherwise non-native β-lactams and to overcome β-lactamase inhibitors (20, 22).

#### Conservation among *bla*_OXA_ variants

**Fig 6:**
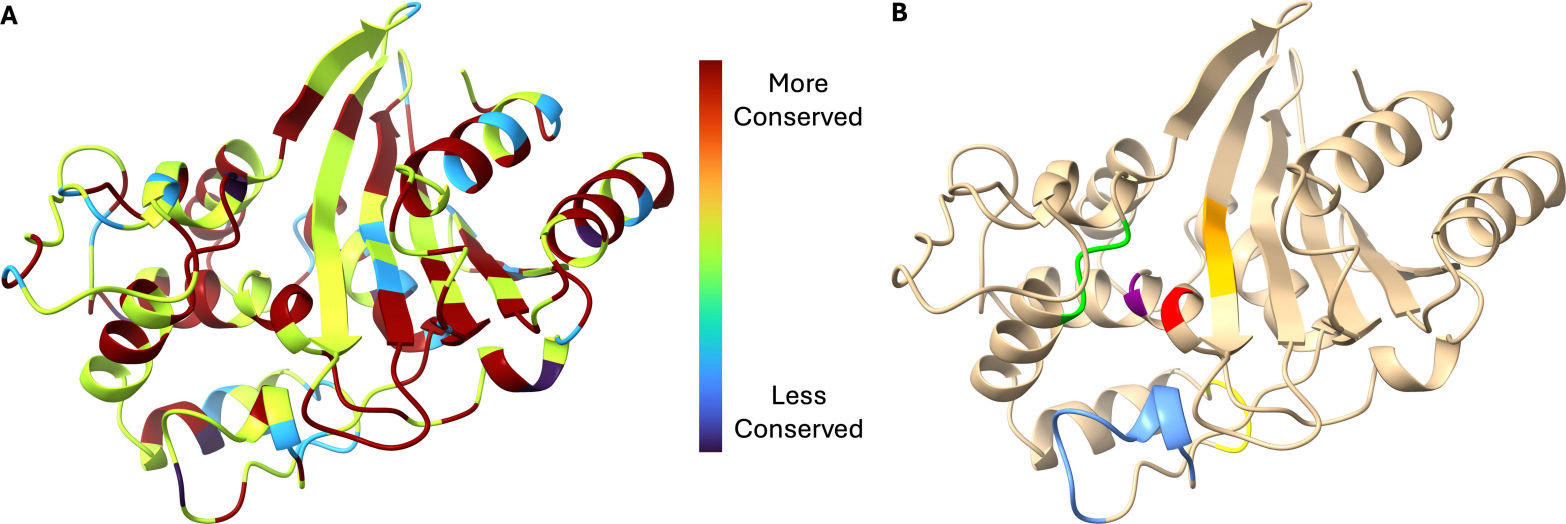
Residue-by-residue conservation of *bla*_OXA-50_ family alleles. (**A**) Color-coded conservation of amino acid residues. (**B**) Important regions and motifs of class D β-lactamases, including the catalytic serine (S70; red), general base lysine (K73; purple), the SXV motif (lime green), the (Y/F)GN motif (yellow), the K(T/S)G motif (golden orange), and the Ω-loop (blue). Visualized on a SWISS-MODEL homology model of OXA-488 based on the crystal structure of OXA-66.

Similarly analyzing *bla*_OXA-50_ family members provides a residue-by-residue overview of amino acid conservation in the *bla*_OXA-50_ family (Fig. 6A). For reference, Fig. 6B highlights important regions and residues of these class D β-lactamases, including the catalytic serine (S70), general base lysine (K73), the SXV motif, the (Y/F)GN motif, the K(T/S)G motif, and the Ω-loop (residues 160–169 by the OXA-66 crystal structure; approximately residues 152–167 by DBL [class D β-lactamase] numbering, some of which are not present in OXA-66) (21, 24, 25).

As anticipated, the essential class D β-lactamase catalytic residues (or biochemical properties of the residues) and characteristic motifs (S70-T71-F72/Y72-K73, S118-X119-V120/I120, Y144-G145-N146, and K216-T217/S217-G218) (21, 26) are highly conserved. The apparently lower conservation of I120 is the result of several amino acids with hydrophobic sidechains being present in that position, likely maintaining many of the properties even as the position becomes somewhat variable.

### Protein sequence identity among *bla*_PDC_ variants

When aligning and examining predicted PDC protein sequences, an interesting observation emerges: the mature proteins of 520 assigned alleles have 98% or higher identity to PDC-3 (the most common PDC variant), 43 have 96%–98% identity, and 5 have 93%–96% identity, while an outlier group of 13 alleles have only 88%–89% identity to PDC-3 but share at least 98% identity with each other, differing by no more than six residues. While the significance of this apparent bifurcation of PDC variants is not immediately clear, one variant (PDC-33) is associated with an isolate previously described as a “taxonomic outlier *Pseudomonas aeruginosa*” (27). Further genetic and biochemical investigation of these variants (and the isolates producing them) is warranted to determine the origin and implications of these outliers.

## DISCUSSION

### Insights into the resistome

Many β-lactamase alleles remain unstudied. While *P. aeruginosa* is a generally well-studied organism, the ratio of distinct assigned to unassigned alleles is surprising, with a total of 622 distinct, unassigned *bla*_PDC_ alleles present in the data set compared to only 254 distinct, assigned alleles, revealing substantial unstudied diversity (as allele assignments are requested by researchers submitting them [28], unassigned alleles are generally unstudied alleles). Interestingly, we only encountered 43.6% of the 582 total assigned *bla*_PDC_ alleles present in the Reference Gene Catalog, further underscoring the rarity of many distinct alleles. This represents a large reservoir of potentially concerning alleles, and understanding and characterizing those with the most resistant phenotypes is an essential area of β-lactamase research.

Carbapenemase prevalence is high and increasing. Carbapenems are broad-spectrum β-lactams that have historically been used as an antibiotic of “last resort” for the treatment of complicated and multidrug-resistant infections (29). As a major concern in MDR *P. aeruginosa* infections, understanding the diversity and prevalence of these enzymes is crucial to overcoming these infections. Disturbingly, the presence of carbapenemases appears to have greatly increased (or carbapenemase-containing isolates have become a bigger proportion of isolates being sequenced and deposited into NCBI databases) over the past two decades. Compared to isolates collected from 2000 to 2012, carbapenemase prevalence is nearly fivefold higher for *P. aeruginosa* isolates collected from 2022 to 2024. Similar observations have been made about increasing carbapenem resistance (30), but further work is needed to better understand the cause of this increase and determine how to best address it.

The distribution of β-lactamase alleles is heavily skewed, and most are uncommon. In 30,452 *P*. *aeruginosa* isolates, we only encountered 254 of 582 assigned *bla*_PDC_ alleles (43.6%). Of these, the majority are extremely uncommon, with 171 *bla*_PDC_ alleles (67.3%) found in 10 or fewer isolates each. Conversely, just five *bla*_PDC_ alleles (*bla*_PDC-3_, *bla*_PDC-5_, *bla*_PDC-8_, *bla*_PDC-1_, and *bla*_PDC-35_) accounted for nearly 60% of all *bla*_PDC_ alleles present.

While investigating the causes of this imbalance remains the territory of future studies, two potential hypotheses are (i) the most common alleles being more ancestral versions of their respective genes, and (ii) purifying selection favoring these variants over others of similar phenotypes. Interestingly, their presence in highly successful clones is likely not sufficient to explain this phenomenon as analysis by both MLST and PDS clusters suggests that they are widespread among very dissimilar isolates.

### Breadth versus depth of allelic diversity

Due to factors including the characteristics of the studies providing sequencing data, outbreaks, and “successful clones,” among others, some closely related groups of isolates are likely overrepresented in the data set. While this may reflect larger trends in the overall population, it runs the risk of masking emerging alleles and deemphasizes alleles arising and disseminating in multiple lineages. In a sense, the “depth” (allele frequency) of diversity overwhelms the “breadth” (number of distinct alleles) of diversity, increasing the odds of important alleles being missed. By deduplicating allele frequencies either by closely related isolates (e.g., MLST and PDS clusters) or isolates collected for a common purpose, often with shared and carefully designed inclusion criteria (BioProject), we can form a better picture of the breadth of allelic diversity without the overshadowing of depth. Interestingly, deduplication by ST, PDS clusters and unclustered isolates, and BioProject all led to generally very minor changes for most alleles, suggesting the overall data set is not greatly skewed as a result and that our analysis is a reasonable approach.

Overall, sequence types are more closely associated with intrinsic *bla*_OXA_ alleles than *bla*_PDC_ alleles, with the latter demonstrating more intra-ST variability (the most widespread ST in the data set, ST235, is associated with nine *bla*_PDC_ alleles but only two intrinsic *bla*_OXA_ alleles across 2,118 isolates). While both sequence types and clusters are typically associated with a small number of alleles, the opposite is not true—many alleles can be found in 10s to 100s of different STs and clusters (the most widespread *bla*_PDC_ allele, *bla*_PDC-3_, is associated with 542 distinct STs and 622 distinct clusters).

Additionally, PDS clusters provide a far more granular picture of isolates than MLST sequence types. The most common sequence type in *P. aeruginosa* corresponds to 244 PDS clusters, while the most common cluster corresponds to just three sequence types. This is not entirely surprising given the much higher resolution associated with the wgMLST origin of PDS clusters, the full extent of this difference is interesting and demonstrates a potential downside of traditional MLST.

### Amino acid conservation

Comparing the sequences of all assigned *bla*_PDC_ and *bla*_OXA-50_ family alleles and corresponding unassigned alleles appearing in this data set, several residues appear less conserved than expected, most notably the catalytic S64 of PDC. Upon closer examination, however, it becomes apparent that the residue is nearly universally conserved with just two variants—PDC-47 and an unassigned PDC— harboring S64L and S64P substitutions, respectively. PDC-47 has only been reported in a single isolate, which the authors expected to be nonfunctional (31). Likewise, we hypothesize that the lack of an essential catalytic residue renders both variants incapable of hydrolysis and unable to contribute to resistance in strains harboring them.

### Comparisons to surveillance studies

The Antibiotic Resistance Leadership Group’s Prospective Observational *Pseudomonas* (POP) study examined CRPa isolates collected from 972 patients across 10 countries in 2018 and 2019 (8). As the POP sequences are available in NCBI databases, 712 isolates belonging to the POP BioProject (PRJNA824880) are included in our study. Collectively, they represent 65 (2.7%) of 2,385 isolates from 2018 and 648 (29.4%) of 2,205 isolates from 2019. These isolates have been excluded from our results for the following comparison and discussion.

POP reported carbapenemase genes in 22% of isolates (8), slightly higher than our findings of 18.5% in 2018 and 2019, but this difference is in line with expectations considering their focus on CRPa. The five most common carbapenemase gene families (*bla*_VIM_, *bla*_NDM_, *bla*_KPC_, *bla*_IMP_, and *bla*_GES_) were the same in POP and our present study, but they found *bla*_KPC_ to be the most common (8). Although defining regions differently, the region containing South America has the highest prevalence of carbapenemase genes in both studies, and the region containing North America is among the lowest (8). We found the most common sequence types to be ST111, ST308, ST463, and ST235, while the POP study found them to be ST235, ST111, ST463, and ST308 ranked differently (8). Overall, many general trends hold true between our work and the POP study, suggesting our methodology is successfully capturing allelic diversity in *P. aeruginosa*.

### Limitations

Although the data set was processed to remove incomplete sequences and “lower-quality” allele calls, some (particularly unassigned) alleles may be inaccurate as the result of sequencing errors. As a genetic and bioinformatic analysis, we did not evaluate protein expression (including derepression and overexpression that can lead to high-level β-lactam resistance in *P. aeruginosa* [32]) or stability, nor the resistance phenotypes of the isolates encoding these alleles. Further studies of individual isolates and enzymes would help to address phenotypic and protein-related questions.

Additionally, while providing a snapshot of allelic diversity across time and place, we have not conducted a surveillance study. In comparison to the more rigid inclusion criteria of a surveillance study, some selection bias exists. The isolates sequenced, submitted, and ultimately used in this analysis were selected based on the needs and interests of the original researchers and studies providing sequencing data, as opposed to a protocol collecting consecutive isolates directly from clinical laboratories in an unbiased manner. Unfortunately, this effect is an intrinsic property of the data set and cannot be readily quantified but is reduced by including as large a sample of isolates as possible.

### Conclusions

In exploring the frequency and distribution of β-lactamase alleles, we have determined important alleles and combinations that warrant coverage by future β-lactams and inhibitors targeting *P. aeruginosa* and that clinicians should be aware of. While this data set is not ideal for examining evolution or selective pressure, we speculate that the existence of a small number of extremely successful enzymes suggests an evolutionary advantage worthy of further study.

With rapidly growing collections of whole genome sequenced bacteria, the NCBI Pathogen Detection Project provides a rich and diverse pool of data on resistance alleles. Querying these data enables exploration of the distribution and diversity of β-lactamase alleles present in *P. aeruginosa* (and, more broadly, the antimicrobial resistance, virulence, and stress response genes in any of the 98 organism groups included in the database). As this data set is diverse in terms of time, geography, and species, we suggest this approach provides useful snapshots of allelic diversity and may serve as an alternative to more traditional, expensive, and time-consuming surveillance studies for examining the distribution and diversity of AMR and related alleles when the epidemiological precision of such a study is not required.

Finally, there remains a large pool of β-lactamase allelic diversity in *P. aeruginosa,* which has largely gone unstudied and unrecognized. Given the high level of plasticity and ease of evolution of β-lactamase enzymes, one can speculate that variants that have not yet faced the right selective pressures could easily spread causing future outbreaks. Ultimately, further microbiological, biochemical, and evolutionary biology studies are needed to truly understand the meaning of this diversity. We assert that understanding the scope and distribution of this diversity is crucial to prioritizing future research and development efforts to focus on the most common and widespread resistance alleles of today and perhaps begin to predict what the future may hold.

## MATERIALS AND METHODS

### Data sources and processing

Data were obtained from databases maintained by the National Center for Biotechnology Information (NCBI), primarily curated as part of the Pathogen Detection Program (https://www.ncbi.nlm.nih.gov/pathogens).

Microbial Browser for Identification of Genetic and Genomic Elements (MicroBIGG-E) (33) data were queried on 9 September 2024 using the Google Cloud Platform interface (https://www.ncbi.nlm.nih.gov/pathogens/docs/microbigge_gcp/) with the query “SELECT * FROM `ncbi-pathogen-detect.pdbrowser.microbigge` WHERE (`scientific_name` like '*Pseudomonas aeruginosa*') AND (`element_symbol` like '%*bla*%').”

Reference Gene Catalog release “2024-07-22.1” (34) was downloaded from the NCBI FTP server (https://ftp.ncbi.nlm.nih.gov/pathogen/Antimicrobial_resistance/Data/2024-07-22.1/ReferenceGeneCatalog.txt) and used to supplement allele and OXA family names as needed.

Identical Protein Group (IPG) accessions were matched to all unassigned alleles and each distinct assigned allele. These accessions were used to group distinct unassigned alleles and update missing allele assignments for alleles added to the Reference Gene Catalog after isolates were processed. IPG identifiers were determined by querying protein accession numbers from MicroBIGG-E using the NCBI Entrez Eutils interface on 9 September 2024 via the REntrez R package.

For purposes of this analysis, alleles are designated as either “assigned” or “unassigned” based on the presence of a formally assigned allele designation. “Assigned” alleles refer to those with a formal designation assigned by NCBI (or Institut Pasteur for *bla*_LEN_, *bla*_OKP-A_, *bla*_OKP-B_, and *bla*_OXY_) and an entry in the Reference Gene Catalog, while “unassigned” alleles refer to those lacking a formal designation and not appearing in the Reference Gene Catalog. Assigned alleles are represented in Pathogen Detection databases with a gene name and number (e.g., *bla*_PDC-3_), while unassigned alleles are represented only by a gene name (e.g., *bla*_PDC_).

To help account for potential sequencing errors and differences in sequence quality (resulting from the use of a large, publicly available data set rather than sequencing and verifying isolates directly), we removed lower-quality alleles (partial alleles with less than 100% coverage, unassigned alleles with less than 90% identity to the closest reference allele, partial alleles crossing contig boundaries, and mistranslations/internal stop codons) from the data set as a final processing step. We also removed alleles identified only by hidden Markov models in AMRFinderPlus (35, 36), as they typically do not contain sufficient detail for a complete analysis. Finally, we removed any isolates not encoding both a *bla*_PDC_ allele and a *bla*_OXA-50_ family member allele, as these are important, intrinsic genes expected in *P. aeruginosa*.

The compiled, processed, and filtered data set used in our analysis has been uploaded to Zenodo as Table S1, available from https://doi.org/10.5281/zenodo.13917360.

### Data analysis

The analysis was performed in RStudio version 2024.04.2 Build 764 using R version 4.4.1.

MLST determinations were made using FastMLST version 0.0.16 (37) with sequences downloaded from NCBI (38). The latest allele and sequence type definitions were downloaded from PubMLST (39) on 10 September 2024.

Original code for downloading, cleaning, analysis, and graphic generation has been prepared as the R package “pdallele.” The exact version used herein is available from https://doi.org/10.5281/zenodo.13917356, and the most current version is available from https://github.com/armack/pdallele/.

### Data notes

The source data use the International Nucleotide Sequence Database Collaboration “country” controlled vocabulary (40), which includes oceans, seas, and several territories and islands as “countries.” As this differs from the definition of “country” used in this analysis, we have processed these country names using the “countrycode” R package, resulting in the dropping of geographic information for ocean/sea locations and some shared or disputed islands from the analysis. Regions (Table S31) are defined based on groupings of United Nations geoscheme subregions (41). We chose to utilize these non-standard regional groupings primarily to provide for the separation of North and South America (which most existing United Nations and World Health Organization regions do not).

Pathogen Detection databases do not explicitly report chromosomal and non-chromosomal alleles for most isolates (i.e., those not assembled at the “complete-genome” level), and as most assemblies do not have chromosomal versus plasmid contigs annotated as such, we assume that *bla*_PDC_ and *bla*_OXA-50_ family alleles are chromosomal, while all other β-lactamase alleles are acquired.

All gene and allele names, *bla*_OXA_ families, and carbapenemase designations are used and discussed as provided in the Pathogen Detection databases.

### Alignments and amino acid conservation

Sequences of all assigned *bla*_PDC_ and *bla*_OXA-50_ family alleles appearing in the Reference Gene Catalog and corresponding unassigned alleles appearing in the MicroBIGG-E data set were downloaded in FASTA format using Identical Protein Group identifiers. Alignments were determined using the MUSCLE3 algorithm (42) with default settings as bundled in UniPro UGENE version 50.0 (43). Amino acid conservation was determined using AL2CO (44) with the default independent counts frequency estimation and entropy-based conservation measure settings as provided in UCSF ChimeraX version 1.8 (45) and used to color code the 4GZB crystal structure of PDC-3 (23) from the RCSB Protein Data Bank (46). Due to the lack of an available crystal structure of an OXA-50 family member, a SWISS-MODEL (47) homology model of OXA-488 was generated based on the 6T1H crystal structure of OXA-66 and used to represent the family.

## SUPPLEMENTAL MATERIAL

Figure S1 aac.00785-24-s0001.pdf Regions used in the analysis and isolate counts for each region.

Supplemental text aac.00785-24-s0002.docx Additional analysis using allele presence data.

Supplemental tables aac.00785-24-s0003.xlsx Tables S2 to S31.

ASM does not own the copyrights to Supplemental Material that may be linked to, or accessed through, an article. The authors have granted ASM a non-exclusive, world-wide license to publish the Supplemental Material files. Please contact the corresponding author directly for reuse.
