# Supplementary material for: β-Lactamase diversity in Pseudomonas aeruginosa
Source: Antimicrob Agents Chemother. 2025 Feb 10;69(3):e00785-24. doi: 10.1128/aac.00785-24 (PMC11881563; doi:10.1128/aac.00785-24)
Supplement: Figure S1 — Regions used in the analysis and isolate counts for each region. [file aac.00785-24-s0001.pdf]

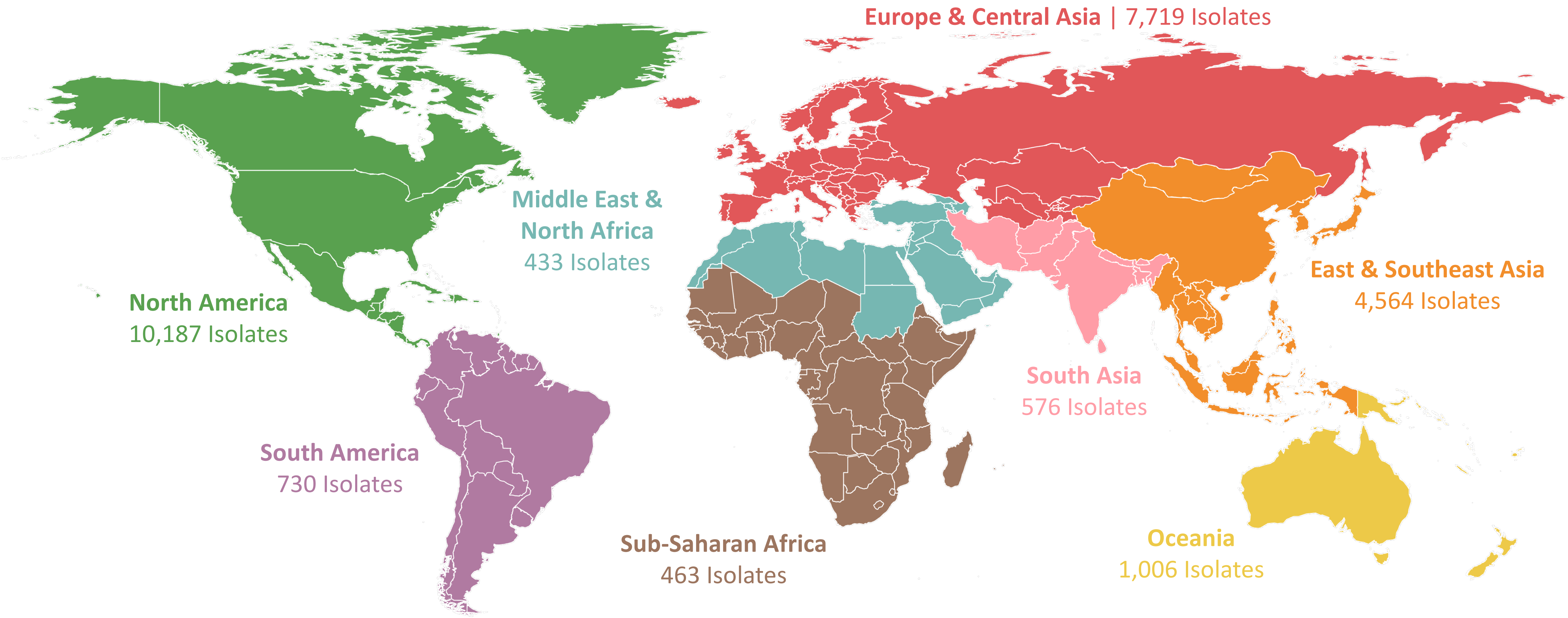

Figure S1: Regions used in the analysis and isolate counts for each region. Regions could not be assigned for 4,774 of 30,452 isolates.
