# Supplementary material for: β-Lactamase diversity in Pseudomonas aeruginosa
Source: Antimicrob Agents Chemother. 2025 Feb 10;69(3):e00785-24. doi: 10.1128/aac.00785-24 (PMC11881563; doi:10.1128/aac.00785-24)
Supplement: Supplemental text — Additional analysis using allele presence data. [file aac.00785-24-s0002.docx]

**Supplemental Material for β-Lactamase Diversity in *Pseudomonas aeruginosa***

Andrew R. Mack, Andrea M. Hujer, Maria F. Mojica, Magdalena A. Taracila, Michael Feldgarden, Daniel H. Haft, William Klimke, Arjun B. Prasad, Robert A. Bonomo

## Overrepresentation of Closely Related Isolates

As a natural consequence of utilizing a data set originally collected for a multitude of different studies as opposed to one collected under a standardized protocol, some closely related isolates are likely overrepresented and may skew the overall results. Herein, we address this effect by examining the binary presence or absence of distinct alleles within groups of closely related isolates. This approach is an effort to mitigate the impact of successful clones and outbreaks while amplifying widespread but low frequency alleles, with the goal of providing a better understanding of the true breadth of allelic diversity in the *P. aeruginosa* β-lactamase resistome. For purposes of comparison, the “greatest differences” refer to alleles moving by at least five ranks and appearing among the 15 most common alleles for at least one set of conditions.

Examining the presence of individual blaPDC alleles by sequence type, the most common are: *bla*_PDC-3_ (542 STs, 30.8%), *bla*_PDC-5_ (367 STs, 20.8%), *bla*_PDC-8_ (113 STs, 6.4%), and *bla*_PDC-1_ (105 STs, 6.0%). Nine additional alleles occur in between 1.0% and 5.0% of STs (**Table S22**). By presence in PDS clusters and unclustered isolates, *bla*_PDC-3_ (3,110 clusters, 20.9%), *bla*_PDC-5_ (2,224 clusters, 15.0%), *bla*_PDC-8_ (1,604 clusters, 10.8%), *bla*_PDC-1_ (1,204 clusters, 8.1%), and *bla*_PDC-35_ (746 clusters, 5.0%) were the most frequent. Eleven additional alleles appeared in between 1.0% and 5.0% of clusters (**Table S23)**. Interestingly, none of the 20 most frequent *bla*_PDC_ alleles shifted in rank by more than two positions when deduplicating by PDS cluster presence while deduplicating by sequence type presence led to several substantial changes.

Compared to the full data set, the greatest differences are: *bla*_PDC-35_ decreasing from fifth most frequent to 13^th^ by MLST, *bla*_PDC-34_ decreasing from eighth most common to 23^rd^ by MLST, *bla*_PDC-11_ decreasing from 10^th^ most common to 15^th^ by MLST, *bla*_PDC-15_ decreasing from 11^th^ most common to 18^th^ by MLST, *bla*_PDC-31_ increasing from 12^th^ most common to sixth by MLST, *bla*_PDC-6_ increasing from 19^th^ most common to 10^th^ by MLST, *bla*_PDC-39_ increasing from 21^st^ most common to 11^th^ by MLST, *bla*_PDC-60_ increasing from 21^st^ most common to 12^th^ by MLST, *bla*_PDC-120_ increasing from 25^th^ most common to 15^th^ by MLST while decreasing to 31^st^ by PDS, *bla*_PDC-66_ increasing from 26^th^ most common to fifth by MLST, *bla*_PDC-97_ increasing from 29^th^ most common to 11^th^ by MLST, *bla*_PDC-45_ increasing from 36^th^ most common to 14^th^ by MLST, *bla*_PDC-23_ increasing from 41^st^ most common to 15^th^ by MLST and 36^th^ by PDS, and *bla*_PDC-51_ increasing from 43^rd^ most common to 15^th^ by MLST and 35^th^ by PDS (**Table S22** and **Table S23**).

Examining the presence of individual *bla*_OXA_ alleles by sequence type, *bla*_OXA-494_ (304 STs, 18.2%), *bla*_OXA-50_ (268 STs, 16.1%), *bla*_OXA-486_ (232 STs, 13.9%), *bla*_OXA-396_ (97 STs, 5.8%), *bla*_OXA-488_ (94 STs, 5.6%), and *bla*_OXA-904_ (89 STs, 5.3%) are the most common. Ten additional alleles occur in between 1.0% and 5.0% of sequence types (**Table S22**). By presence in PDS clusters and unclustered isolates, the most frequent alleles are: *bla*_OXA-50_ (2,303 clusters, 15.1%), *bla*_OXA-488_ (2,093 clusters, 13.7%), *bla*_OXA-494_ (1,995 clusters, 13.1%), *bla*_OXA-486_ (1,839 clusters, 12.0%), *bla*_OXA-396_ (1,165 clusters, 7.6%), *bla*_OXA-395_ (913 clusters, 6.0%), and *bla*_OXA-487_ (810 clusters, 5.3%). Eight additional alleles occur in between 1.0% and 5.0% of isolates (**Table S23**).

Compared the full data set, the greatest differences are: *bla*_OXA-905_ decreasing from ninth most frequent to 16^th^ by MLST, *bla*_OXA-846_ decreasing from 10^th^ most common to 18^th^ by MLST, *bla*_OXA-848_ decreasing from 11^th^ most common to 19^th^ by MLST, *bla*_OXA-1035_ decreasing from 15^th^ most common to 26^th^ by MLST, *bla*_OXA-1028_ increasing from 13^th^ most common to eighth by MLST, *bla*_OXA-1127_ increasing from 17^th^ most common to 11^th^ by MLST, *bla*_OXA-1014_ increasing from 21^st^ most common to 13^th^ by MLST, *bla*_OXA-1135_ increasing from 25^th^ most common to 14^th^ by MLST, *bla*_OXA-1029_ increasing from 29^th^ most common to 14^th^ by MLST, and *bla*_OXA-902_ increasing from 30^th^ most common to 14^th^ by MLST (**Table S22** and **Table S23**).
